# Supplementary material for: Male reproductive traits are differentially affected by dietary macronutrient balance but unrelated to adiposity
Source: Nat Commun. 2023 May 4;14:2566. doi: 10.1038/s41467-023-38314-x (PMC10160019; doi:10.1038/s41467-023-38314-x)
Supplement: Supplementary file 1 — Supplementary Information [file 41467_2023_38314_MOESM1_ESM.pdf]

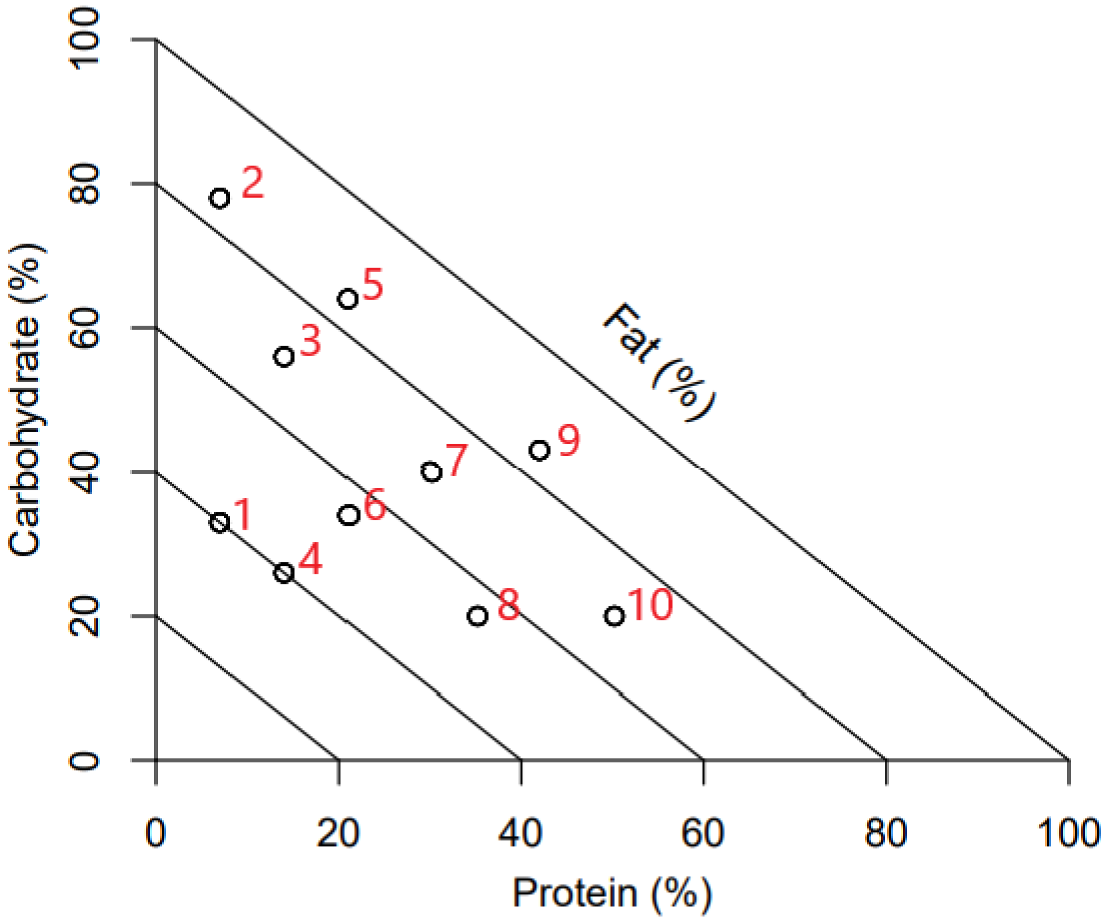

Supplementary Figure 1. The 10 isocaloric experimental diets represented in space on a right-angled mixture triangle. Macronutrients are given as the percentage of total kcal, with fat represented on the implicit axis (hypotenuse) and increasing in value towards the origin. Diet compositions are specified in Table I

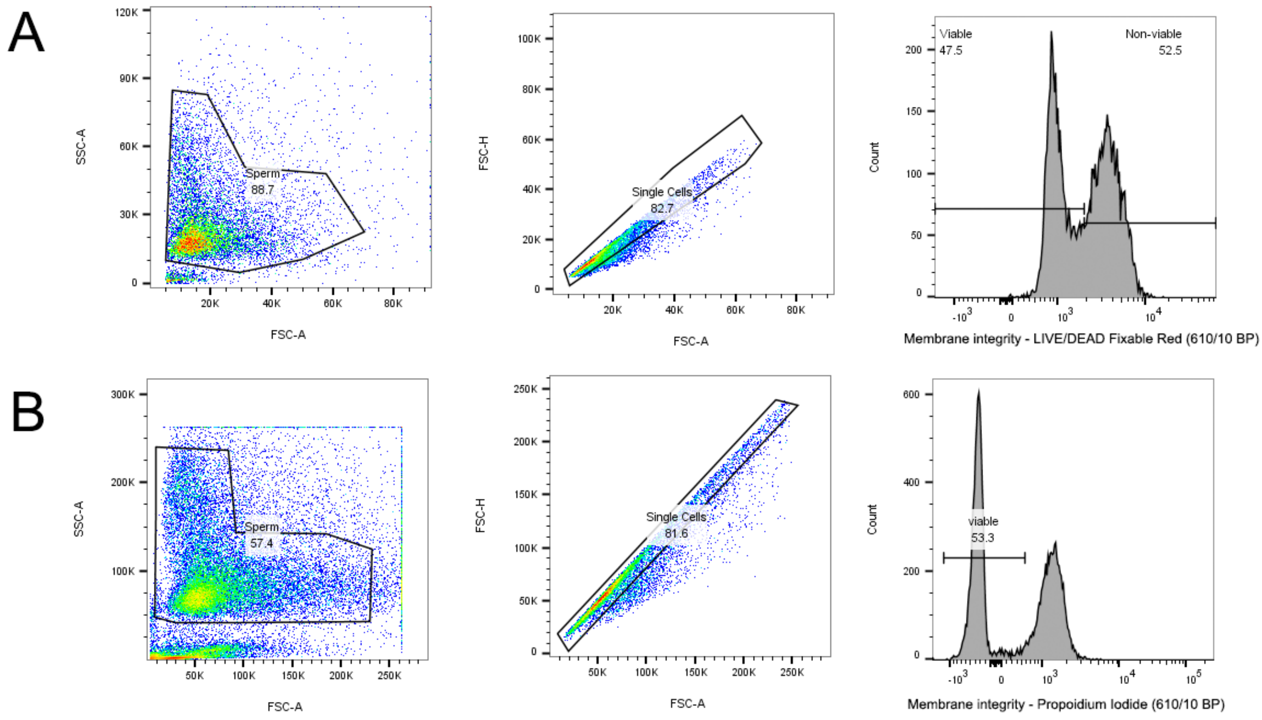

Supplementary Figure 2. Gating strategy for flow cytometry. All samples were gated firstly on the basis of forward and side scatter to isolate spermatozoa from debris (left hand panels), and subsequently on the basis of forward scatter area and height to isolate single cells (middle panels). For the fixable viability stain (A), a 610/10 BP histogram was used to determine the proportion of viable (unstained) spermatozoa (right hand panel). For H2DCFDA (B), the population of single cells was further gated based on a 610/10 BP histogram to discriminate the viable (PI negative) population and subsequently measure median 525/50 BP detector fluorescence (right hand panel).

Supplementary Table 1. Primers used for qPCR analysis of testicular gene expression

Gene	Source	Forward primer	Reverse primer	PCR product size
ACTB	Own design	CGCAGCCACTGTCGAGTC	ATCCATGGCGAACTGGTGG	93
ADH5	Kicqstart	CAGAAAATAAGGGTCACTCAG	GGATCGATTTTAGCAACAGAG	152
CAT	Kicqstart	CTCCATCAGGTTTCTTTCTTG	CAACAGGCAAGTTTTTGATG	166
GPX1	Kicqstart	GGAGAATGGCAAGAATGAAG	TTCGCACTTCTCAAACAATG	97
GPX4	Kicqstart	TGGATAAGTACAGGGGTTTC	TAGCTGAGTGTAGTTTACGTC	83
GSR	Kicqstart	GTTCACAGGTTAAGGAAG	TATTCAGATTCAGGCCCTTAG	162
GSS	Kicqstart	TATGGATGACCAGGAAGTTG	CCCAGTTCTGTGAATTATACTG	77
HSD17B3	Kicqstart	ATCATTATTCAGGTGCTGAC	GGATCCGGTTCAGAATTATTG	181
NRF2 (NFE2L2)	Kicqstart	CATTCCCGAATTACAGTGTC	GGAGATCGATGAGTAAAAATGG	124
RPLP0	Kicqstart	GTTCTCCTATAAAAGGCACAC	AAAGTTGGATGATCTTGAGG	172
SOD1	Own design	GGGAAGCATGGCGATGAAAG	GGTTCACCGCTTGCCTTCTG	93
SOD2	Kicqstart	CCATTTTCTGGACAAACCTG	GACCTTGCTCCTTATTGAAG	177
SOD3	Kicqstart	AGAGAGAGTATTTGGGAACC	AAACTAAGCTGCAAAGTCTC	104
STAR	Kicqstart	GCGGAATATGAAAGGATTAAGG	GTCACTATAGAGTGTTGCTTC	157
TFRC	Kicqstart	CAGAAGTTTCTGGTAAACTGG	TCTGCAAAAGTAATTTCCCC	121

3150 Great Eastern Hwy
Glen Forrest
Western Australia 6071
p: +61 8 9298 8111
F: +61 8 9298 8700
Email: info@specialtyfeeds.com

Diet

SF19-076

Diet 1b: 7P 60F 33C Energy modification of AIN93G

A semi-pure diet formulation for laboratory rats and mice based on AIN-93G. Some modifications have been made to the original formulation to suit locally available raw materials.

- An omega 3 to omega 6 fatty acid ratio has been set at 1:3.7. Lipids are from Soybean oil, lard and linseed oil.
- Protein is from a protein mix specified by customer made from Casein, Whey Protein Isolate and supplemented Leucine, Threonine, Methionine, Tyrosine, Phenylalanine, Tryptophan, Alanine, Aspartic Acid, Arginine, Glycine, Histidine and Serine.
- Net metabolisable energy has been set to 14.7 MJ/Kg
- Cellulose inclusion has been allowed to float to act as a filler
- This diet does not meet minimum amino acid requirements as described by Gahl et al 1991, The Journal of Nutrition and reported in NRC guidelines Nutrient Requirements for Laboratory Animals.
- See Calculated Nutritional Parameters for energy break down

Calculated Nutritional Parameters

Protein	6.7%
Total Fat	24.0%
Crude Fibre	29.8%
AD Fibre	29.8%
Total Carbohydrate	30.2%
Net Metabolisable Energy	14.7 MJ / Kg
% Total calculated net metabolisable energy from lipids	60.0%
% Total calculated net metabolisable energy from Carbohydrates	33.0%
% Total calculated net metabolisable energy from protein	7.0%

Diet Form and Features

- Semi pure diet. 12 mm diameter pellets.
- Pack size 1.5 Kg, vacuum packed in oxygen impermeable plastic bags, under nitrogen. Bags are packed into cardboard cartons to protect them during transit. Smaller pack quantity on request.
- Diet suitable for irradiation but not suitable for autoclave.
- Lead time 2 weeks for non-irradiation or 4 weeks for irradiation.

Ingredients	
Protein Mix	71 g/Kg
Sucrose	51 g/Kg
Soybean Oil	150 g/Kg
Lard	70 g/Kg
Linseed Oil	22 g/Kg
Cellulose	320 g/Kg
Wheat Starch	208 g/Kg
Dextrinised Starch	68 g/Kg
Calcium Carbonate	13.1 g/Kg
Sodium Chloride	2.6 g/Kg
AIN93 Trace Minerals	1.4 g/Kg
Potassium Citrate	2.5 g/Kg
Potassium Dihydrogen Phosphate	6.9 g/Kg
Potassium Sulphate	1.6 g/Kg
Choline Chloride (75%)	2.5 g/Kg
AIN93 Vitamins	10 g/Kg

Calculated Total Vitamins as Fed	
Vitamin A (Retinol)	4 000 IU/Kg
Vitamin D (Cholecalciferol)	1 000 IU/Kg
Vitamin E (a Tocopherol acetate)	81 mg/Kg
Vitamin K (Menadione)	1 mg/Kg
Vitamin C (Ascorbic acid)	None added
Vitamin B1 (Thiamine)	6.0 mg/Kg
Vitamin B2 (Riboflavin)	6.0 mg/Kg
Niacin (Nicotinic acid)	30 mg/Kg
Vitamin B6 (Pryridoxine)	7 mg/Kg
Pantothenic Acid	16 mg/Kg
Biotin	200 ug/Kg
Folic Acid	2 mg/Kg
Inositol	None added
Vitamin B12 (Cyancobalamin)	100 ug/Kg
Choline	2 020 mg/Kg

Calculated Amino Acids as Fed		
	Recommended Minimum	Level in Diet
Valine	0.74%	0.34%
Leucine	1.07%	0.65%
Isoleucine	0.62%	0.30%
Threonine	0.62%	0.39%
Methionine*	0.98%	0.24%
Cysteine		0.07%
Lysine	0.92%	0.43%
Phenylalanine°	1.02%	0.26%
Tyrosine		0.26%
Tryptophan	0.20%	0.11%
Alanine		0.30%
Aspartic Acid		0.38%
Glutamic Acid		0.59%
Proline		0.44%
Asparagine		0.19%
Glutamine		0.42%
Arginine	0.48%	0.51%
Glycine		0.23%
Histidine	0.28%	0.19%
Serine		0.42%

*One half of L-Methionine can be replaced with L-Cystine
°One Half of L-Phenalalanine can be replaced with L-Tyrosine

Calculated Total Minerals as Fed	
Calcium	0.69%
Phosphorous	0.21%
Magnesium	0.06%
Sodium	0.15%
Chloride	0.16%
Potassium	0.41%
Sulphur	0.11%
Iron	50 mg/Kg
Copper	8.5 mg/Kg
Iodine	0.2 mg/Kg
Manganese	22 mg/Kg
Cobalt	No data
Zinc	40 mg/Kg
Molybdenum	0.15 mg/Kg
Selenium	0.2 mg/Kg
Cadmium	No data
Chromium	1.0 mg/Kg
Fluoride	1.0 mg/Kg
Lithium	0.1 mg/Kg
Boron	1.2 mg/Kg
Nickel	0.5 mg/Kg
Vanadium	0.1 mg/Kg

Calculated Fatty Acid Composition as Fed	
Saturated Fats C12:0 and less	0.02%
Myristic Acid 14:0	0.12%
Palmitic Acid 16:0	3.50%
Stearic Acid 18:0	1.84%
Palmitoleic Acid 16:1	0.15%
Oleic Acid 18:1	6.13%
Gadoleic Acid 20:1	0.08%
Linoleic Acid 18:2 n6	8.95%
a Linolenic Acid 18:3 n3	2.40%
Arachadonic Acid 20:4 n6	No data
EPA 20:5 n3	No data
DHA 22:6 n3	No data
Total n3	2.41%
Total n6	8.96%
Total Mono Unsaturated Fats	6.39%
Total Polyunsaturated Fats	11.41%
Total Saturated Fats	5.57%

Calculated data uses information from typical raw material composition. It could be expected that individual batches of diet will vary from this figure. **Diet post treatment by irradiation or autoclave could change these parameters**. We are happy to provide full calculated nutritional information for all of our products, however we would like to emphasise that these diets have been specifically designed for manufacture by Specialty Feeds.

3150 Great Eastern Hwy
Glen Forrest
Western Australia 6071
p: +61 8 9298 8111
F: +61 8 9298 8700
Email: info@specialtyfeeds.com

Diet

SF19-077

Diet 2b: 7P 15F 78C Energy modification of AIN93G

A semi-pure diet formulation for laboratory rats and mice based on AIN-93G. Some modifications have been made to the original formulation to suit locally available raw materials.

- An omega 3 to omega 6 fatty acid ratio has been set at 1:3.7. Lipids are from Soybean oil, lard and linseed oil.
- Protein is from a protein mix specified by customer made from Casein, Whey Protein Isolate and supplemented Leucine, Threonine, Methionine, Tyrosine, Phenylalanine, Tryptophan, Alanine, Aspartic Acid, Arginine, Glycine, Histidine and Serine.
- Net metabolisable energy has been set to 14.7 MJ/Kg
- Cellulose inclusion has been allowed to float to act as a filler
- This diet does not meet minimum amino acid requirements as described by Gahl et al 1991, The Journal of Nutrition and reported in NRC guidelines Nutrient Requirements for Laboratory Animals.
- See Calculated Nutritional Parameters for energy break down

Calculated Nutritional Parameters

Protein	6.7%
Total Fat	6.0%
Crude Fibre	3.7%
AD Fibre	3.7%
Total Carbohydrate	71.3%
Net Metabolisable Energy	14.7 MJ / Kg
% Total calculated net metabolisable energy from lipids	15.0%
% Total calculated net metabolisable energy from Carbohydrates	78.0%
% Total calculated net metabolisable energy from protein	7.0%

Diet Form and Features

- Semi pure diet. 12 mm diameter pellets.
- Pack size 5 Kg, vacuum packed in oxygen impermeable plastic bags, under nitrogen. Bags are packed into cardboard cartons to protect them during transit. Smaller pack quantity on request.
- Diet suitable for irradiation but not suitable for autoclave.
- Lead time 2 weeks for non-irradiation or 4 weeks for irradiation.

Ingredients	
Protein Mix	71 g/Kg
Sucrose	124 g/Kg
Soybean Oil	37 g/Kg
Lard	17.5 g/Kg
Linseed Oil	5.4 g/Kg
Cellulose	40 g/Kg
Wheat Starch	501 g/Kg
Dextrinised Starch	163 g/Kg
Calcium Carbonate	13.1 g/Kg
Sodium Chloride	2.6 g/Kg
AIN93 Trace Minerals	1.4 g/Kg
Potassium Citrate	2.5 g/Kg
Potassium Dihydrogen Phosphate	6.9 g/Kg
Potassium Sulphate	1.6 g/Kg
Choline Chloride (75%)	2.5 g/Kg
AIN93 Vitamins	10 g/Kg

Calculated Total Vitamins as Fed	
Vitamin A (Retinol)	4 000 IU/Kg
Vitamin D (Cholecalciferol)	1 000 IU/Kg
Vitamin E (a Tocopherol acetate)	77 mg/Kg
Vitamin K (Menadione)	1 mg/Kg
Vitamin C (Ascorbic acid)	None added
Vitamin B1 (Thiamine)	6.0 mg/Kg
Vitamin B2 (Riboflavin)	6.0 mg/Kg
Niacin (Nicotinic acid)	30 mg/Kg
Vitamin B6 (Pryridoxine)	7 mg/Kg
Pantothenic Acid	16 mg/Kg
Biotin	200 ug/Kg
Folic Acid	2 mg/Kg
Inositol	None added
Vitamin B12 (Cyancobalamin)	100 ug/Kg
Choline	2 150 mg/Kg

Calculated Amino Acids as Fed		
	Recommended Minimum	Level in Diet
Valine	0.74%	0.34%
Leucine	1.07%	0.65%
Isoleucine	0.62%	0.30%
Threonine	0.62%	0.39%
Methionine*	0.98%	0.24%
Cysteine		0.07%
Lysine	0.92%	0.43%
Phenylalanine°	1.02%	0.26%
Tyrosine		0.26%
Tryptophan	0.20%	0.11%
Alanine		0.30%
Aspartic Acid		0.38%
Glutamic Acid		0.59%
Proline		0.44%
Asparagine		0.19%
Glutamine		0.42%
Arginine	0.48%	0.51%
Glycine		0.23%
Histidine	0.28%	0.19%
Serine		0.42%

*One half of L-Methionine can be replaced with L-Cystine
°One Half of L-Phenalalanine can be replaced with L-Tyrosine

Calculated Total Minerals as Fed	
Calcium	0.69%
Phosphorous	0.24%
Magnesium	0.06%
Sodium	0.15%
Chloride	0.16%
Potassium	0.41%
Sulphur	0.12%
Iron	50 mg/Kg
Copper	6.9 mg/Kg
Iodine	0.2 mg/Kg
Manganese	16 mg/Kg
Cobalt	No data
Zinc	40 mg/Kg
Molybdenum	0.15 mg/Kg
Selenium	0.2 mg/Kg
Cadmium	No data
Chromium	1.0 mg/Kg
Fluoride	1.0 mg/Kg
Lithium	0.1 mg/Kg
Boron	2.0 mg/Kg
Nickel	0.5 mg/Kg
Vanadium	0.1 mg/Kg

Calculated Fatty Acid Composition as Fed	
Saturated Fats C12:0 and less	0.01%
Myristic Acid 14:0	0.03%
Palmitic Acid 16:0	0.88%
Stearic Acid 18:0	0.46%
Palmitoleic Acid 16:1	0.04%
Oleic Acid 18:1	1.53%
Gadoleic Acid 20:1	0.02%
Linoleic Acid 18:2 n6	2.24%
a Linolenic Acid 18:3 n3	0.60%
Arachadonic Acid 20:4 n6	No data
EPA 20:5 n3	No data
DHA 22:6 n3	No data
Total n3	0.60%
Total n6	2.24%
Total Mono Unsaturated Fats	1.6%
Total Polyunsaturated Fats	2.85%
Total Saturated Fats	1.39%

Calculated data uses information from typical raw material composition. It could be expected that individual batches of diet will vary from this figure. **Diet post treatment by irradiation or autoclave could change these parameters**. We are happy to provide full calculated nutritional information for all of our products, however we would like to emphasise that these diets have been specifically designed for manufacture by Specialty Feeds.

3150 Great Eastern Hwy
Glen Forrest
Western Australia 6071
p: +61 8 9298 8111
F: +61 8 9298 8700
Email: info@specialtyfeeds.com

Diet SF19-078	Diet 3b: 14P 30F 56C Energy modification of AIN93G

A semi-pure diet formulation for laboratory rats and mice based on AIN-93G. Some modifications have been made to the original formulation to suit locally available raw materials.

- An omega 3 to omega 6 fatty acid ratio has been set at 1:3.7. Lipids are from Soybean oil, lard and linseed oil.
- Protein is from a protein mix specified by customer made from Casein, Whey Protein Isolate and supplemented Leucine, Threonine, Methionine, Tyrosine, Phenylalanine, Tryptophan, Alanine, Aspartic Acid, Arginine, Glycine, Histidine and Serine.
- Net metabolisable energy has been set to 14.7 MJ/Kg
- Cellulose inclusion has been allowed to float to act as a filler
- This diet does not meet minimum amino acid requirements as described by Gahl et al 1991, The Journal of Nutrition and reported in NRC guidelines Nutrient Requirements for Laboratory Animals.
- See Calculated Nutritional Parameters for energy break down

Calculated Nutritional Parameters	
Protein	13.5%
Total Fat	12.0%
Crude Fibre	12.4%
AD Fibre	12.4%
Total Carbohydrate	51.3%
Net Metabolisable Energy	14.7 MJ / Kg
% Total calculated net metabolisable energy from lipids	30.0%
% Total calculated net metabolisable energy from Carbohydrates	56.0%
% Total calculated net metabolisable energy from protein	14.0%

Diet Form and Features

- Semi pure diet. 12 mm diameter pellets.
- Pack size 5 Kg, vacuum packed in oxygen impermeable plastic bags, under nitrogen. Bags are packed into cardboard cartons to protect them during transit. Smaller pack quantity on request.
- Diet suitable for irradiation but not suitable for autoclave.
- Lead time 2 weeks for non-irradiation or 4 weeks for irradiation.

Ingredients

Protein Mix	142 g/Kg
Sucrose	88 g/Kg
Soybean Oil	75 g/Kg
Lard	35 g/Kg
Linseed Oil	11 g/Kg
Cellulose	134 g/Kg
Wheat Starch	358 g/Kg
Dextrinised Starch	117 g/Kg
Calcium Carbonate	13.1 g/Kg
Sodium Chloride	2.6 g/Kg
AIN93 Trace Minerals	1.4 g/Kg
Potassium Citrate	2.5 g/Kg
Potassium Dihydrogen Phosphate	6.9 g/Kg
Potassium Sulphate	1.6 g/Kg
Choline Chloride (75%)	2.5 g/Kg
AIN93 Vitamins	10 g/Kg

Calculated Total Vitamins as Fed

Vitamin A (Retinol)	4 000 IU/Kg
Vitamin D (Cholecalciferol)	1 000 IU/Kg
Vitamin E (a Tocopherol acetate)	78 mg/Kg
Vitamin K (Menadione)	1 mg/Kg
Vitamin C (Ascorbic acid)	None added
Vitamin B1 (Thiamine)	6.0 mg/Kg
Vitamin B2 (Riboflavin)	6.0 mg/Kg
Niacin (Nicotinic acid)	30 mg/Kg
Vitamin B6 (Pryridoxine)	7 mg/Kg
Pantothenic Acid	16 mg/Kg
Biotin	200 ug/Kg
Folic Acid	2 mg/Kg
Inositol	None added
Vitamin B12 (Cyancobalamin)	101 ug/Kg
Choline	2 100 mg/Kg

Calculated Amino Acids as Fed

	Recommended Minimum	Level in Diet
Valine	0.74%	0.67%
Leucine	1.07%	1.30%
Isoleucine	0.62%	0.59%
Threonine	0.62%	0.78%
Methionine*	0.98%	0.48%
Cysteine		0.15%
Lysine	0.92%	0.86%
Phenylalanine°	1.02%	0.53%
Tyrosine		0.51%
Tryptophan	0.20%	0.23%
Alanine		0.60%
Aspartic Acid		0.76%
Glutamic Acid		1.18%
Proline		0.87%
Asparagine		0.38%
Glutamine		0.84%
Arginine	0.48%	1.03%
Glycine		0.47%
Histidine	0.28%	0.38%
Serine		0.83%

*One half of L-Methionine can be replaced with L-Cystine
°One Half of L-Phenalalanine can be replaced with L-Tyrosine

Calculated Total Minerals as Fed	
Calcium	0.69%
Phosphorous	0.25%
Magnesium	0.06%
Sodium	0.15%
Chloride	0.16%
Potassium	0.44%
Sulphur	0.18%
Iron	50 mg/Kg
Copper	7.4 mg/Kg
Iodine	0.2 mg/Kg
Manganese	18 mg/Kg
Cobalt	No data
Zinc	41 mg/Kg
Molybdenum	0.15 mg/Kg
Selenium	0.3 mg/Kg
Cadmium	No data
Chromium	1.0 mg/Kg
Fluoride	1.0 mg/Kg
Lithium	0.1 mg/Kg
Boron	1.8 mg/Kg
Nickel	0.5 mg/Kg
Vanadium	0.1 mg/Kg

Calculated Fatty Acid Composition as Fed	
Saturated Fats C12:0 and less	0.01%
Myristic Acid 14:0	0.06%
Palmitic Acid 16:0	1.75%
Stearic Acid 18:0	0.92%
Palmitoleic Acid 16:1	0.08%
Oleic Acid 18:1	3.06%
Gadoleic Acid 20:1	0.04%
Linoleic Acid 18:2 n6	4.48%
a Linolenic Acid 18:3 n3	1.20%
Arachadonic Acid 20:4 n6	No data
EPA 20:5 n3	No data
DHA 22:6 n3	No data
Total n3	1.21%
Total n6	4.48%
Total Mono Unsaturated Fats	3.19%
Total Polyunsaturated Fats	5.70%
Total Saturated Fats	2.78%

Calculated data uses information from typical raw material composition. It could be expected that individual batches of diet will vary from this figure. **Diet post treatment by irradiation or autoclave could change these parameters**. We are happy to provide full calculated nutritional information for all of our products, however we would like to emphasise that these diets have been specifically designed for manufacture by Specialty Feeds.

3150 Great Eastern Hwy
Glen Forrest
Western Australia 6071
p: +61 8 9298 8111
F: +61 8 9298 8700
Email: info@specialtyfeeds.com

Diet

SF19-079

Diet 4b: 14P 60F 26C Energy modification of AIN93G

A semi-pure diet formulation for laboratory rats and mice based on AIN-93G. Some modifications have been made to the original formulation to suit locally available raw materials.

- An omega 3 to omega 6 fatty acid ratio has been set at 1:3.7. Lipids are from Soybean oil, lard and linseed oil.
- Protein is from a protein mix specified by customer made from Casein, Whey Protein Isolate and supplemented Leucine, Threonine, Methionine, Tyrosine, Phenylalanine, Tryptophan, Alanine, Aspartic Acid, Arginine, Glycine, Histidine and Serine.
- Net metabolisable energy has been set to 14.7 MJ/Kg
- Cellulose inclusion has been allowed to float to act as a filler
- This diet does not meet minimum amino acid requirements as described by Gahl et al 1991, The Journal of Nutrition and reported in NRC guidelines Nutrient Requirements for Laboratory Animals.
- See Calculated Nutritional Parameters for energy break down

Calculated Nutritional Parameters

Protein	13.5%
Total Fat	24.0%
Crude Fibre	29.8%
AD Fibre	29.8%
Total Carbohydrate	23.8%
Net Metabolisable Energy	14.7 MJ / Kg
% Total calculated net metabolisable energy from lipids	60.0%
% Total calculated net metabolisable energy from Carbohydrates	26.0%
% Total calculated net metabolisable energy from protein	14.0%

Diet Form and Features

- Semi pure diet. 12 mm diameter pellets.
- Pack size 1.5 Kg, vacuum packed in oxygen impermeable plastic bags, under nitrogen. Bags are packed into cardboard cartons to protect them during transit. Smaller pack quantity on request.
- Diet suitable for irradiation but not suitable for autoclave.
- Lead time 2 weeks for non-irradiation or 4 weeks for irradiation.

Ingredients

Protein Mix	142 g/Kg
Sucrose	40 g/Kg
Soybean Oil	150 g/Kg
Lard	70 g/Kg
Linseed Oil	21.5 g/Kg
Cellulose	320 g/Kg
Wheat Starch	163 g/Kg
Dextrinised Starch	53 g/Kg
Calcium Carbonate	13.1 g/Kg
Sodium Chloride	2.6 g/Kg
AIN93 Trace Minerals	1.4 g/Kg
Potassium Citrate	2.5 g/Kg
Potassium Dihydrogen Phosphate	6.9 g/Kg
Potassium Sulphate	1.6 g/Kg
Choline Chloride (75%)	2.5 g/Kg
AIN93 Vitamins	10 g/Kg

Calculated Total Vitamins as Fed

Vitamin A (Retinol)	4 000 IU/Kg
Vitamin D (Cholecalciferol)	1 000 IU/Kg
Vitamin E (a Tocopherol acetate)	81 mg/Kg
Vitamin K (Menadione)	1 mg/Kg
Vitamin C (Ascorbic acid)	None added
Vitamin B1 (Thiamine)	6.0 mg/Kg
Vitamin B2 (Riboflavin)	6.0 mg/Kg
Niacin (Nicotinic acid)	30 mg/Kg
Vitamin B6 (Pryridoxine)	7 mg/Kg
Pantothenic Acid	16 mg/Kg
Biotin	200 ug/Kg
Folic Acid	2 mg/Kg
Inositol	None added
Vitamin B12 (Cyancobalamin)	101 ug/Kg
Choline	2 010 mg/Kg

Calculated Amino Acids as Fed

	Recommended Minimum	Level in Diet
Valine	0.74%	0.67%
Leucine	1.07%	1.30%
Isoleucine	0.62%	0.59%
Threonine	0.62%	0.78%
Methionine*	0.98%	0.48%
Cysteine		0.15%
Lysine	0.92%	0.86%
Phenylalanine°	1.02%	0.53%
Tyrosine		0.51%
Tryptophan	0.20%	0.23%
Alanine		0.60%
Aspartic Acid		0.76%
Glutamic Acid		1.18%
Proline		0.87%
Asparagine		0.38%
Glutamine		0.84%
Arginine	0.48%	1.03%
Glycine		0.47%
Histidine	0.28%	0.38%
Serine		0.83%

*One half of L-Methionine can be replaced with L-Cystine
°One Half of L-Phenalalanine can be replaced with L-Tyrosine

Calculated Total Minerals as Fed	
Calcium	0.69%
Phosphorous	0.24%
Magnesium	0.06%
Sodium	0.14%
Chloride	0.16%
Potassium	0.44%
Sulphur	0.18%
Iron	50 mg/Kg
Copper	8.5 mg/Kg
Iodine	0.2 mg/Kg
Manganese	22 mg/Kg
Cobalt	No data
Zinc	41 mg/Kg
Molybdenum	0.15 mg/Kg
Selenium	0.3 mg/Kg
Cadmium	No data
Chromium	1.0 mg/Kg
Fluoride	1.0 mg/Kg
Lithium	0.1 mg/Kg
Boron	1.3 mg/Kg
Nickel	0.5 mg/Kg
Vanadium	0.1 mg/Kg

Calculated Fatty Acid Composition as Fed	
Saturated Fats C12:0 and less	0.02%
Myristic Acid 14:0	0.12%
Palmitic Acid 16:0	3.50%
Stearic Acid 18:0	1.84%
Palmitoleic Acid 16:1	0.15%
Oleic Acid 18:1	6.13%
Gadoleic Acid 20:1	0.08%
Linoleic Acid 18:2 n6	8.95%
a Linolenic Acid 18:3 n3	2.40%
Arachadonic Acid 20:4 n6	No data
EPA 20:5 n3	No data
DHA 22:6 n3	No data
Total n3	2.41%
Total n6	8.96%
Total Mono Unsaturated Fats	6.39%
Total Polyunsaturated Fats	11.41%
Total Saturated Fats	5.57%

Calculated data uses information from typical raw material composition. It could be expected that individual batches of diet will vary from this figure. **Diet post treatment by irradiation or autoclave could change these parameters**. We are happy to provide full calculated nutritional information for all of our products, however we would like to emphasise that these diets have been specifically designed for manufacture by Specialty Feeds.

3150 Great Eastern Hwy
Glen Forrest
Western Australia 6071
p: +61 8 9298 8111
F: +61 8 9298 8700
Email: info@specialtyfeeds.com

Diet SF19-080 — Diet 5b: 21P 15F 64C Energy modification of AIN93G

A semi-pure diet formulation for laboratory rats and mice based on AIN-93G. Some modifications have been made to the original formulation to suit locally available raw materials.

- An omega 3 to omega 6 fatty acid ratio has been set at 1:3.7. Lipids are from Soybean oil, lard and linseed oil.
- Protein is from a protein mix specified by customer made from Casein, Whey Protein Isolate and supplemented Leucine, Threonine, Methionine, Tyrosine, Phenylalanine, Tryptophan, Alanine, Aspartic Acid, Arginine, Glycine, Histidine and Serine.
- Net metabolisable energy has been set to 14.7 MJ/Kg
- Cellulose inclusion has been allowed to float to act as a filler
- See Calculated Nutritional Parameters for energy break down

Calculated Nutritional Parameters

Protein	20.2%
Total Fat	6.0%
Crude Fibre	3.8%
AD Fibre	3.8%
Total Carbohydrate	58.6%
Net Metabolisable Energy	14.7 MJ / Kg
% Total calculated net metabolisable energy from lipids	15.0%
% Total calculated net metabolisable energy from Carbohydrates	64.0%
% Total calculated net metabolisable energy from protein	21.0%

Diet Form and Features

- Semi pure diet. 12 mm diameter pellets.
- Pack size 5 Kg, vacuum packed in oxygen impermeable plastic bags, under nitrogen. Bags are packed into cardboard cartons to protect them during transit. Smaller pack quantity on request.
- Diet suitable for irradiation but not suitable for autoclave.
- Lead time 2 weeks for non-irradiation or 4 weeks for irradiation.

Ingredients	
Protein Mix	213 g/Kg
Sucrose	101 g/Kg
Soybean Oil	37 g/Kg
Lard	17.5 g/Kg
Linseed Oil	5.4 g/Kg
Cellulose	41 g/Kg
Wheat Starch	410 g/Kg
Dextrinised Starch	134 g/Kg
Calcium Carbonate	13.1 g/Kg
Sodium Chloride	2.6 g/Kg
AIN93 Trace Minerals	1.4 g/Kg
Potassium Citrate	2.5 g/Kg
Potassium Dihydrogen Phosphate	6.9 g/Kg
Potassium Sulphate	1.6 g/Kg
Choline Chloride (75%)	2.5 g/Kg
AIN93 Vitamins	10 g/Kg

Calculated Total Vitamins as Fed	
Vitamin A (Retinol)	4 000 IU/Kg
Vitamin D (Cholecalciferol)	1 000 IU/Kg
Vitamin E (a Tocopherol acetate)	77 mg/Kg
Vitamin K (Menadione)	1 mg/Kg
Vitamin C (Ascorbic acid)	None added
Vitamin B1 (Thiamine)	6.0 mg/Kg
Vitamin B2 (Riboflavin)	6.1 mg/Kg
Niacin (Nicotinic acid)	30 mg/Kg
Vitamin B6 (Pryridoxine)	7 mg/Kg
Pantothenic Acid	16 mg/Kg
Biotin	200 ug/Kg
Folic Acid	2 mg/Kg
Inositol	None added
Vitamin B12 (Cyancobalamin)	101 ug/Kg
Choline	2 130 mg/Kg

Calculated Amino Acids as Fed		
	Recommended Minimum	Level in Diet
Valine	0.74%	1.01%
Leucine	1.07%	1.95%
Isoleucine	0.62%	0.89%
Threonine	0.62%	1.17%
Methionine*	0.98%	0.72%
Cysteine		0.22%
Lysine	0.92%	1.30%
Phenylalanine°	1.02%	0.79%
Tyrosine		0.77%
Tryptophan	0.20%	0.34%
Alanine		0.90%
Aspartic Acid		1.15%
Glutamic Acid		1.77%
Proline		1.31%
Asparagine		0.57%
Glutamine		1.25%
Arginine	0.48%	1.54%
Glycine		0.70%
Histidine	0.28%	0.57%
Serine		1.25%

*One half of L-Methionine can be replaced with L-Cystine
°One Half of L-Phenalalanine can be replaced with L-Tyrosine

Calculated Total Minerals as Fed	
Calcium	0.69%
Phosphorous	0.28%
Magnesium	0.06%
Sodium	0.15%
Chloride	0.16%
Potassium	0.46%
Sulphur	0.25%
Iron	50 mg/Kg
Copper	6.9 mg/Kg
Iodine	0.2 mg/Kg
Manganese	16 mg/Kg
Cobalt	No data
Zinc	42 mg/Kg
Molybdenum	0.15 mg/Kg
Selenium	0.3 mg/Kg
Cadmium	No data
Chromium	1.0 mg/Kg
Fluoride	1.0 mg/Kg
Lithium	0.1 mg/Kg
Boron	2.1 mg/Kg
Nickel	0.5 mg/Kg
Vanadium	0.1 mg/Kg

Calculated Fatty Acid Composition as Fed	
Saturated Fats C12:0 and less	0.01%
Myristic Acid 14:0	0.03%
Palmitic Acid 16:0	0.88%
Stearic Acid 18:0	0.46%
Palmitoleic Acid 16:1	0.04%
Oleic Acid 18:1	1.53%
Gadoleic Acid 20:1	0.02%
Linoleic Acid 18:2 n6	2.24%
a Linolenic Acid 18:3 n3	0.60%
Arachadonic Acid 20:4 n6	No data
EPA 20:5 n3	No data
DHA 22:6 n3	No data
Total n3	0.60%
Total n6	2.24%
Total Mono Unsaturated Fats	1.6%
Total Polyunsaturated Fats	2.85%
Total Saturated Fats	1.39%

Calculated data uses information from typical raw material composition. It could be expected that individual batches of diet will vary from this figure. **Diet post treatment by irradiation or autoclave could change these parameters**. We are happy to provide full calculated nutritional information for all of our products, however we would like to emphasise that these diets have been specifically designed for manufacture by Specialty Feeds.

3150 Great Eastern Hwy
Glen Forrest
Western Australia 6071
p: +61 8 9298 8111
F: +61 8 9298 8700
Email: info@specialtyfeeds.com

Diet

SF19-081

Diet 6b: 21P 45F 34C Energy modification of AIN93G

A semi-pure diet formulation for laboratory rats and mice based on AIN-93G. Some modifications have been made to the original formulation to suit locally available raw materials.

- An omega 3 to omega 6 fatty acid ratio has been set at 1:3.7. Lipids are from Soybean oil, lard and linseed oil.
- Protein is from a protein mix specified by customer made from Casein, Whey Protein Isolate and supplemented Leucine, Threonine, Methionine, Tyrosine, Phenylalanine, Tryptophan, Alanine, Aspartic Acid, Arginine, Glycine, Histidine and Serine.
- Net metabolisable energy has been set to 14.7 MJ/Kg
- Cellulose inclusion has been allowed to float to act as a filler

- See Calculated Nutritional Parameters for energy break down

Calculated Nutritional Parameters

Protein	20.2%
Total Fat	18.0%
Crude Fibre	21.2%
AD Fibre	21.2%
Total Carbohydrate	31.2%
Net Metabolisable Energy	14.7 MJ / Kg
% Total calculated net metabolisable energy from lipids	45.0%
% Total calculated net metabolisable energy from Carbohydrates	34.0%
% Total calculated net metabolisable energy from protein	21.0%

Diet Form and Features

- Semi pure diet. 12 mm diameter pellets.
- Pack size 1.5 Kg, vacuum packed in oxygen impermeable plastic bags, under nitrogen. Bags are packed into cardboard cartons to protect them during transit. Smaller pack quantity on request.
- Diet suitable for irradiation but not suitable for autoclave.
- Lead time 2 weeks for non-irradiation or 4 weeks for irradiation.

Ingredients	
Protein Mix	213 g/Kg
Sucrose	53 g/Kg
Soybean Oil	112 g/Kg
Lard	53 g/Kg
Linseed Oil	16 g/Kg
Cellulose	227 g/Kg
Wheat Starch	215 g/Kg
Dextrinised Starch	70 g/Kg
Calcium Carbonate	13.1 g/Kg
Sodium Chloride	2.6 g/Kg
AIN93 Trace Minerals	1.4 g/Kg
Potassium Citrate	2.5 g/Kg
Potassium Dihydrogen Phosphate	6.9 g/Kg
Potassium Sulphate	1.6 g/Kg
Choline Chloride (75%)	2.5 g/Kg
AIN93 Vitamins	10 g/Kg

Calculated Total Vitamins as Fed	
Vitamin A (Retinol)	4 000 IU/Kg
Vitamin D (Cholecalciferol)	1 000 IU/Kg
Vitamin E (a Tocopherol acetate)	80 mg/Kg
Vitamin K (Menadione)	1 mg/Kg
Vitamin C (Ascorbic acid)	None added
Vitamin B1 (Thiamine)	6.0 mg/Kg
Vitamin B2 (Riboflavin)	6.1 mg/Kg
Niacin (Nicotinic acid)	30 mg/Kg
Vitamin B6 (Pryridoxine)	7 mg/Kg
Pantothenic Acid	16 mg/Kg
Biotin	200 ug/Kg
Folic Acid	2 mg/Kg
Inositol	None added
Vitamin B12 (Cyancobalamin)	101 ug/Kg
Choline	2 040 mg/Kg

Calculated Amino Acids as Fed	Recommended Minimum	Level in Diet
Valine	0.74%	1.01%
Leucine	1.07%	1.95%
Isoleucine	0.62%	0.89%
Threonine	0.62%	1.17%
Methionine*	0.98%	0.72%
Cysteine		0.22%
Lysine	0.92%	1.30%
Phenylalanine°	1.02%	0.79%
Tyrosine		0.77%
Tryptophan	0.20%	0.34%
Alanine		0.90%
Aspartic Acid		1.15%
Glutamic Acid		1.77%
Proline		1.31%
Asparagine		0.57%
Glutamine		1.25%
Arginine	0.48%	1.54%
Glycine		0.70%
Histidine	0.28%	0.57%
Serine		1.25%

*One half of L-Methionine can be replaced with L-Cystine
°One Half of L-Phenalalanine can be replaced with L-Tyrosine

Calculated Total Minerals as Fed	
Calcium	0.69%
Phosphorous	0.27%
Magnesium	0.06%
Sodium	0.15%
Chloride	0.16%
Potassium	0.46%
Sulphur	0.24%
Iron	50 mg/Kg
Copper	8.0 mg/Kg
Iodine	0.2 mg/Kg
Manganese	20 mg/Kg
Cobalt	No data
Zinc	42 mg/Kg
Molybdenum	0.15 mg/Kg
Selenium	0.3 mg/Kg
Cadmium	No data
Chromium	1.0 mg/Kg
Fluoride	1.0 mg/Kg
Lithium	0.1 mg/Kg
Boron	1.6 mg/Kg
Nickel	0.5 mg/Kg
Vanadium	0.1 mg/Kg

Calculated Fatty Acid Composition as Fed	
Saturated Fats C12:0 and less	0.02%
Myristic Acid 14:0	0.09%
Palmitic Acid 16:0	2.63%
Stearic Acid 18:0	1.38%
Palmitoleic Acid 16:1	0.11%
Oleic Acid 18:1	4.60%
Gadoleic Acid 20:1	0.06%
Linoleic Acid 18:2 n6	6.71%
a Linolenic Acid 18:3 n3	1.80%
Arachadonic Acid 20:4 n6	No data
EPA 20:5 n3	No data
DHA 22:6 n3	No data
Total n3	1.81%
Total n6	6.72%
Total Mono Unsaturated Fats	4.79%
Total Polyunsaturated Fats	8.55%
Total Saturated Fats	4.17%

Calculated data uses information from typical raw material composition. It could be expected that individual batches of diet will vary from this figure. **Diet post treatment by irradiation or autoclave could change these parameters**. We are happy to provide full calculated nutritional information for all of our products, however we would like to emphasise that these diets have been specifically designed for manufacture by Specialty Feeds.

3150 Great Eastern Hwy
Glen Forrest
Western Australia 6071
p: +61 8 9298 8111
F: +61 8 9298 8700
Email: info@specialtyfeeds.com

Diet SF19-082 — Diet 7b: 30P 30F 40C Energy modification of AIN93G

A semi-pure diet formulation for laboratory rats and mice based on AIN-93G. Some modifications have been made to the original formulation to suit locally available raw materials.

- An omega 3 to omega 6 fatty acid ratio has been set at 1:3.7. Lipids are from Soybean oil, lard and linseed oil.
- Protein is from a protein mix specified by customer made from Casein, Whey Protein Isolate and supplemented Leucine, Threonine, Methionine, Tyrosine, Phenylalanine, Tryptophan, Alanine, Aspartic Acid, Arginine, Glycine, Histidine and Serine.
- Net metabolisable energy has been set to 14.7 MJ/Kg
- Cellulose inclusion has been allowed to float to act as a filler

- See Calculated Nutritional Parameters for energy break down

Calculated Nutritional Parameters

Protein	28.9%
Total Fat	12.0%
Crude Fibre	12.6%
AD Fibre	12.6%
Total Carbohydrate	36.7%
Net Metabolisable Energy	14.7 MJ / Kg
% Total calculated net metabolisable energy from lipids	30.0%
% Total calculated net metabolisable energy from Carbohydrates	40.0%
% Total calculated net metabolisable energy from protein	30.0%

Diet Form and Features

- Semi pure diet. 12 mm diameter pellets.
- Pack size 1.5 Kg, vacuum packed in oxygen impermeable plastic bags, under nitrogen. Bags are packed into cardboard cartons to protect them during transit. Smaller pack quantity on request.
- Diet suitable for irradiation but not suitable for autoclave.
- Lead time 2 weeks for non-irradiation or 4 weeks for irradiation.

Ingredients

Protein Mix	305 g/Kg
Sucrose	63 g/Kg
Soybean Oil	75 g/Kg
Lard	35 g/Kg
Linseed Oil	11 g/Kg
Cellulose	135 g/Kg
Wheat Starch	254 g/Kg
Dextrinised Starch	83 g/Kg
Calcium Carbonate	13.1 g/Kg
Sodium Chloride	2.6 g/Kg
AIN93 Trace Minerals	1.4 g/Kg
Potassium Citrate	2.5 g/Kg
Potassium Dihydrogen Phosphate	6.9 g/Kg
Potassium Sulphate	1.6 g/Kg
Choline Chloride (75%)	2.5 g/Kg
AIN93 Vitamins	10 g/Kg

Calculated Total Vitamins as Fed

Vitamin A (Retinol)	4 000 IU/Kg
Vitamin D (Cholecalciferol)	1 000 IU/Kg
Vitamin E (a Tocopherol acetate)	78 mg/Kg
Vitamin K (Menadione)	1 mg/Kg
Vitamin C (Ascorbic acid)	None added
Vitamin B1 (Thiamine)	6.0 mg/Kg
Vitamin B2 (Riboflavin)	6.2 mg/Kg
Niacin (Nicotinic acid)	30 mg/Kg
Vitamin B6 (Pryridoxine)	7 mg/Kg
Pantothenic Acid	16 mg/Kg
Biotin	200 ug/Kg
Folic Acid	2 mg/Kg
Inositol	None added
Vitamin B12 (Cyancobalamin)	102 ug/Kg
Choline	2 070 mg/Kg

Calculated Amino Acids as Fed

	Recommended Minimum	Level in Diet
Valine	0.74%	1.45%
Leucine	1.07%	2.79%
Isoleucine	0.62%	1.27%
Threonine	0.62%	1.68%
Methionine*	0.98%	1.02%
Cysteine		0.33%
Lysine	0.92%	1.85%
Phenylalanine°	1.02%	1.13%
Tyrosine		1.10%
Tryptophan	0.20%	0.49%
Alanine		1.29%
Aspartic Acid		1.64%
Glutamic Acid		2.53%
Proline		1.87%
Asparagine		0.82%
Glutamine		1.79%
Arginine	0.48%	2.20%
Glycine		1.00%
Histidine	0.28%	0.81%
Serine		1.78%

*One half of L-Methionine can be replaced with L-Cystine
°One Half of L-Phenalalanine can be replaced with L-Tyrosine

Calculated Total Minerals as Fed	
Calcium	0.69%
Phosphorous	0.30%
Magnesium	0.06%
Sodium	0.15%
Chloride	0.16%
Potassium	0.49%
Sulphur	0.33%
Iron	50 mg/Kg
Copper	7.5 mg/Kg
Iodine	0.2 mg/Kg
Manganese	18 mg/Kg
Cobalt	No data
Zinc	44 mg/Kg
Molybdenum	0.15 mg/Kg
Selenium	0.4 mg/Kg
Cadmium	No data
Chromium	1.0 mg/Kg
Fluoride	1.0 mg/Kg
Lithium	0.1 mg/Kg
Boron	1.9 mg/Kg
Nickel	0.5 mg/Kg
Vanadium	0.1 mg/Kg

Calculated Fatty Acid Composition as Fed	
Saturated Fats C12:0 and less	0.01%
Myristic Acid 14:0	0.06%
Palmitic Acid 16:0	1.75%
Stearic Acid 18:0	0.92%
Palmitoleic Acid 16:1	0.08%
Oleic Acid 18:1	3.06%
Gadoleic Acid 20:1	0.04%
Linoleic Acid 18:2 n6	4.48%
a Linolenic Acid 18:3 n3	1.20%
Arachadonic Acid 20:4 n6	No data
EPA 20:5 n3	No data
DHA 22:6 n3	No data
Total n3	1.21%
Total n6	4.48%
Total Mono Unsaturated Fats	3.19%
Total Polyunsaturated Fats	5.70%
Total Saturated Fats	2.78%

Calculated data uses information from typical raw material composition. It could be expected that individual batches of diet will vary from this figure. **Diet post treatment by irradiation or autoclave could change these parameters**. We are happy to provide full calculated nutritional information for all of our products, however we would like to emphasise that these diets have been specifically designed for manufacture by Specialty Feeds.

3150 Great Eastern Hwy
Glen Forrest
Western Australia 6071
p: +61 8 9298 8111
F: +61 8 9298 8700
Email: info@specialtyfeeds.com

Diet

SF19-083

Diet 8b: 35P 45F 20C Energy modification of AIN93G

A semi-pure diet formulation for laboratory rats and mice based on AIN-93G. Some modifications have been made to the original formulation to suit locally available raw materials.

- An omega 3 to omega 6 fatty acid ratio has been set at 1:3.7. Lipids are from Soybean oil, lard and linseed oil.
- Protein is from a protein mix specified by customer made from Casein, Whey Protein Isolate and supplemented Leucine, Threonine, Methionine, Tyrosine, Phenylalanine, Tryptophan, Alanine, Aspartic Acid, Arginine, Glycine, Histidine and Serine.
- Net metabolisable energy has been set to 14.7 MJ/Kg
- Cellulose inclusion has been allowed to float to act as a filler

- See Calculated Nutritional Parameters for energy break down

Calculated Nutritional Parameters	
Protein	33.7%
Total Fat	18.0%
Crude Fibre	21.3%
AD Fibre	21.3%
Total Carbohydrate	18.4%
Net Metabolisable Energy	14.7 MJ / Kg
% Total calculated net metabolisable energy from lipids	45.0%
% Total calculated net metabolisable energy from Carbohydrates	20.0%
% Total calculated net metabolisable energy from protein	35.0%

Diet Form and Features

- Semi pure diet. 12 mm diameter pellets.
- Pack size 1.5 Kg, vacuum packed in oxygen impermeable plastic bags, under nitrogen. Bags are packed into cardboard cartons to protect them during transit. Smaller pack quantity on request.
- Diet suitable for irradiation but not suitable for autoclave.
- Lead time 2 weeks for non-irradiation or 4 weeks for irradiation.

Ingredients	
Protein Mix	356 g/Kg
Sucrose	31 g/Kg
Soybean Oil	112 g/Kg
Lard	53 g/Kg
Linseed Oil	16 g/Kg
Cellulose	229 g/Kg
Wheat Starch	124 g/Kg
Dextrinised Starch	83 g/Kg
Calcium Carbonate	13.1 g/Kg
Sodium Chloride	2.6 g/Kg
AIN93 Trace Minerals	1.4 g/Kg
Potassium Citrate	2.5 g/Kg
Potassium Dihydrogen Phosphate	6.9 g/Kg
Potassium Sulphate	1.6 g/Kg
Choline Chloride (75%)	2.5 g/Kg
AIN93 Vitamins	10 g/Kg

Calculated Total Vitamins as Fed	
Vitamin A (Retinol)	4 000 IU/Kg
Vitamin D (Cholecalciferol)	1 000 IU/Kg
Vitamin E (a Tocopherol acetate)	80 mg/Kg
Vitamin K (Menadione)	1 mg/Kg
Vitamin C (Ascorbic acid)	None added
Vitamin B1 (Thiamine)	6.0 mg/Kg
Vitamin B2 (Riboflavin)	6.2 mg/Kg
Niacin (Nicotinic acid)	30 mg/Kg
Vitamin B6 (Pryridoxine)	7 mg/Kg
Pantothenic Acid	16 mg/Kg
Biotin	200 ug/Kg
Folic Acid	2 mg/Kg
Inositol	None added
Vitamin B12 (Cyancobalamin)	102 ug/Kg
Choline	2 020 mg/Kg

Calculated Amino Acids as Fed	Recommended Minimum	Level in Diet
Valine	0.74%	1.69%
Leucine	1.07%	3.25%
Isoleucine	0.62%	1.48%
Threonine	0.62%	1.96%
Methionine*	0.98%	1.19%
Cysteine		0.38%
Lysine	0.92%	2.16%
Phenylalanine°	1.02%	1.32%
Tyrosine		1.29%
Tryptophan	0.20%	0.57%
Alanine		1.51%
Aspartic Acid		1.91%
Glutamic Acid		2.95%
Proline		2.18%
Asparagine		0.96%
Glutamine		2.09%
Arginine	0.48%	2.56%
Glycine		1.17%
Histidine	0.28%	0.95%
Serine		2.08%

*One half of L-Methionine can be replaced with L-Cystine
°One Half of L-Phenalalanine can be replaced with L-Tyrosine

Calculated Total Minerals as Fed	
Calcium	0.70%
Phosphorous	0.31%
Magnesium	0.06%
Sodium	0.14%
Chloride	0.16%
Potassium	0.50%
Sulphur	0.37%
Iron	50 mg/Kg
Copper	8.0 mg/Kg
Iodine	0.2 mg/Kg
Manganese	20 mg/Kg
Cobalt	No data
Zinc	44 mg/Kg
Molybdenum	0.15 mg/Kg
Selenium	0.4 mg/Kg
Cadmium	No data
Chromium	1.0 mg/Kg
Fluoride	1.0 mg/Kg
Lithium	0.1 mg/Kg
Boron	1.7 mg/Kg
Nickel	0.5 mg/Kg
Vanadium	0.1 mg/Kg

Calculated Fatty Acid Composition as Fed	
Saturated Fats C12:0 and less	0.02%
Myristic Acid 14:0	0.09%
Palmitic Acid 16:0	2.63%
Stearic Acid 18:0	1.38%
Palmitoleic Acid 16:1	0.11%
Oleic Acid 18:1	4.60%
Gadoleic Acid 20:1	0.06%
Linoleic Acid 18:2 n6	6.71%
a Linolenic Acid 18:3 n3	1.80%
Arachadonic Acid 20:4 n6	No data
EPA 20:5 n3	No data
DHA 22:6 n3	No data
Total n3	1.81%
Total n6	6.72%
Total Mono Unsaturated Fats	4.79%
Total Polyunsaturated Fats	8.55%
Total Saturated Fats	4.17%

Calculated data uses information from typical raw material composition. It could be expected that individual batches of diet will vary from this figure. **Diet post treatment by irradiation or autoclave could change these parameters**. We are happy to provide full calculated nutritional information for all of our products, however we would like to emphasise that these diets have been specifically designed for manufacture by Specialty Feeds.

3150 Great Eastern Hwy
Glen Forrest
Western Australia 6071
p: +61 8 9298 8111
F: +61 8 9298 8700
Email: info@specialtyfeeds.com

Diet

SF19-084

Diet 9b: 42P 15F 43C Energy modification of AIN93G

A semi-pure diet formulation for laboratory rats and mice based on AIN-93G. Some modifications have been made to the original formulation to suit locally available raw materials.

- An omega 3 to omega 6 fatty acid ratio has been set at 1:3.7. Lipids are from Soybean oil, lard and linseed oil.
- Protein is from a protein mix specified by customer made from Casein, Whey Protein Isolate and supplemented Leucine, Threonine, Methionine, Tyrosine, Phenylalanine, Tryptophan, Alanine, Aspartic Acid, Arginine, Glycine, Histidine and Serine.
- Net metabolisable energy has been set to 14.7 MJ/Kg
- Cellulose inclusion has been allowed to float to act as a filler
- See Calculated Nutritional Parameters for energy break down

Calculated Nutritional Parameters	
Protein	40.4%
Total Fat	6.0%
Crude Fibre	3.9%
AD Fibre	3.9%
Total Carbohydrate	39.5%
Net Metabolisable Energy	14.7 MJ / Kg
% Total calculated net metabolisable energy from lipids	15.0%
% Total calculated net metabolisable energy from Carbohydrates	43.0%
% Total calculated net metabolisable energy from protein	42.0%

Diet Form and Features

- Semi pure diet. 12 mm diameter pellets.
- Pack size 5 Kg, vacuum packed in oxygen impermeable plastic bags, under nitrogen. Bags are packed into cardboard cartons to protect them during transit. Smaller pack quantity on request.
- Diet suitable for irradiation but not suitable for autoclave.
- Lead time 2 weeks for non-irradiation or 4 weeks for irradiation.

Ingredients	
Protein Mix	427 g/Kg
Sucrose	68 g/Kg
Soybean Oil	37 g/Kg
Lard	17.5 g/Kg
Linseed Oil	5.4 g/Kg
Cellulose	42 g/Kg
Wheat Starch	274 g/Kg
Dextrinised Starch	89 g/Kg
Calcium Carbonate	13.1 g/Kg
Sodium Chloride	2.6 g/Kg
AIN93 Trace Minerals	1.4 g/Kg
Potassium Citrate	2.5 g/Kg
Potassium Dihydrogen Phosphate	6.9 g/Kg
Potassium Sulphate	1.6 g/Kg
Choline Chloride (75%)	2.5 g/Kg
AIN93 Vitamins	10 g/Kg

Calculated Total Vitamins as Fed	
Vitamin A (Retinol)	4 000 IU/Kg
Vitamin D (Cholecalciferol)	1 000 IU/Kg
Vitamin E (a Tocopherol acetate)	77 mg/Kg
Vitamin K (Menadione)	1 mg/Kg
Vitamin C (Ascorbic acid)	None added
Vitamin B1 (Thiamine)	6.0 mg/Kg
Vitamin B2 (Riboflavin)	6.3 mg/Kg
Niacin (Nicotinic acid)	30 mg/Kg
Vitamin B6 (Pryridoxine)	7 mg/Kg
Pantothenic Acid	16 mg/Kg
Biotin	200 ug/Kg
Folic Acid	2 mg/Kg
Inositol	None added
Vitamin B12 (Cyancobalamin)	103 ug/Kg
Choline	2 100 mg/Kg

Calculated Amino Acids as Fed		
	Recommended Minimum	Level in Diet
Valine	0.74%	2.02%
Leucine	1.07%	3.90%
Isoleucine	0.62%	1.78%
Threonine	0.62%	2.35%
Methionine*	0.98%	1.43%
Cysteine		0.45%
Lysine	0.92%	2.59%
Phenylalanine°	1.02%	1.58%
Tyrosine		1.55%
Tryptophan	0.20%	0.69%
Alanine		1.81%
Aspartic Acid		1.29%
Glutamic Acid		3.54%
Proline		2.61%
Asparagine		1.15%
Glutamine		2.51%
Arginine	0.48%	3.07%
Glycine		1.40%
Histidine	0.28%	1.14%
Serine		2.49%

*One half of L-Methionine can be replaced with L-Cystine
°One Half of L-Phenalalanine can be replaced with L-Tyrosine

Calculated Total Minerals as Fed	
Calcium	0.70%
Phosphorous	0.35%
Magnesium	0.06%
Sodium	0.14%
Chloride	0.16%
Potassium	0.53%
Sulphur	0.44%
Iron	50 mg/Kg
Copper	6.9 mg/Kg
Iodine	0.2 mg/Kg
Manganese	16 mg/Kg
Cobalt	No data
Zinc	45 mg/Kg
Molybdenum	0.15 mg/Kg
Selenium	0.4 mg/Kg
Cadmium	No data
Chromium	1.0 mg/Kg
Fluoride	1.0 mg/Kg
Lithium	0.1 mg/Kg
Boron	2.3 mg/Kg
Nickel	0.5 mg/Kg
Vanadium	0.1 mg/Kg

Calculated Fatty Acid Composition as Fed	
Saturated Fats C12:0 and less	0.01%
Myristic Acid 14:0	0.03%
Palmitic Acid 16:0	0.88%
Stearic Acid 18:0	0.46%
Palmitoleic Acid 16:1	0.04%
Oleic Acid 18:1	1.53%
Gadoleic Acid 20:1	0.02%
Linoleic Acid 18:2 n6	2.24%
a Linolenic Acid 18:3 n3	0.60%
Arachadonic Acid 20:4 n6	No data
EPA 20:5 n3	No data
DHA 22:6 n3	No data
Total n3	0.60%
Total n6	2.24%
Total Mono Unsaturated Fats	1.60%
Total Polyunsaturated Fats	2.85%
Total Saturated Fats	1.39%

Calculated data uses information from typical raw material composition. It could be expected that individual batches of diet will vary from this figure. **Diet post treatment by irradiation or autoclave could change these parameters**. We are happy to provide full calculated nutritional information for all of our products, however we would like to emphasise that these diets have been specifically designed for manufacture by Specialty Feeds.

3150 Great Eastern Hwy
Glen Forrest
Western Australia 6071
p: +61 8 9298 8111
F: +61 8 9298 8700
Email: info@specialtyfeeds.com

Diet

SF19-085

Diet 10b: 50P 30F 20C Energy modification of AIN93G

A semi-pure diet formulation for laboratory rats and mice based on AIN-93G. Some modifications have been made to the original formulation to suit locally available raw materials.

- An omega 3 to omega 6 fatty acid ratio has been set at 1:3.7. Lipids are from Soybean oil, lard and linseed oil.
- Protein is from a protein mix specified by customer made from Casein, Whey Protein Isolate and supplemented Leucine, Threonine, Methionine, Tyrosine, Phenylalanine, Tryptophan, Alanine, Aspartic Acid, Arginine, Glycine, Histidine and Serine.
- Net metabolisable energy has been set to 14.7 MJ/Kg
- Cellulose inclusion has been allowed to float to act as a filler

- See Calculated Nutritional Parameters for energy break down

Calculated Nutritional Parameters

Protein	48.1%
Total Fat	12.1%
Crude Fibre	12.7%
AD Fibre	12.7%
Total Carbohydrate	18.5%
Net Metabolisable Energy	14.7 MJ / Kg
% Total calculated net metabolisable energy from lipids	30.0%
% Total calculated net metabolisable energy from Carbohydrates	20.0%
% Total calculated net metabolisable energy from protein	50.0%

Diet Form and Features

- Semi pure diet. 12 mm diameter pellets.
- Pack size 1.5 Kg, vacuum packed in oxygen impermeable plastic bags, under nitrogen. Bags are packed into cardboard cartons to protect them during transit. Smaller pack quantity on request.
- Diet suitable for irradiation but not suitable for autoclave.
- Lead time 2 weeks for non-irradiation or 4 weeks for irradiation.

Ingredients	
Protein Mix	508 g/Kg
Sucrose	31 g/Kg
Soybean Oil	75 g/Kg
Lard	35 g/Kg
Linseed Oil	11 g/Kg
Cellulose	136 g/Kg
Wheat Starch	124 g/Kg
Dextrinised Starch	40 g/Kg
Calcium Carbonate	13.1 g/Kg
Sodium Chloride	2.6 g/Kg
AIN93 Trace Minerals	1.4 g/Kg
Potassium Citrate	2.5 g/Kg
Potassium Dihydrogen Phosphate	6.9 g/Kg
Potassium Sulphate	1.6 g/Kg
Choline Chloride (75%)	2.5 g/Kg
AIN93 Vitamins	10 g/Kg

Calculated Total Vitamins as Fed	
Vitamin A (Retinol)	4 000 IU/Kg
Vitamin D (Cholecalciferol)	1 000 IU/Kg
Vitamin E (a Tocopherol acetate)	78 mg/Kg
Vitamin K (Menadione)	1 mg/Kg
Vitamin C (Ascorbic acid)	None added
Vitamin B1 (Thiamine)	6.0 mg/Kg
Vitamin B2 (Riboflavin)	6.3 mg/Kg
Niacin (Nicotinic acid)	30 mg/Kg
Vitamin B6 (Pryridoxine)	7 mg/Kg
Pantothenic Acid	16.6 mg/Kg
Biotin	200 ug/Kg
Folic Acid	2 mg/Kg
Inositol	None added
Vitamin B12 (Cyancobalamin)	103 ug/Kg
Choline	2 040 mg/Kg

Calculated Amino Acids as Fed	Recommended Minimum	Level in Diet
Valine	0.74%	2.41%
Leucine	1.07%	4.65%
Isoleucine	0.62%	2.12%
Threonine	0.62%	2.78%
Methionine*	0.98%	1.70%
Cysteine		0.54%
Lysine	0.92%	3.09%
Phenylalanine°	1.02%	1.88%
Tyrosine		1.84%
Tryptophan	0.20%	0.82%
Alanine		2.15%
Aspartic Acid		2.73%
Glutamic Acid		4.21%
Proline		3.11%
Asparagine		1.37%
Glutamine		2.98%
Arginine	0.48%	3.66%
Glycine		1.66%
Histidine	0.28%	1.36%
Serine		2.96%

*One half of L-Methionine can be replaced with L-Cystine
°One Half of L-Phenalalanine can be replaced with L-Tyrosine

Calculated Total Minerals as Fed	
Calcium	0.70%
Phosphorous	0.37%
Magnesium	0.06%
Sodium	0.15%
Chloride	0.16%
Potassium	0.55%
Sulphur	0.52%
Iron	50 mg/Kg
Copper	7.5 mg/Kg
Iodine	0.2 mg/Kg
Manganese	17 mg/Kg
Cobalt	No data
Zinc	46 mg/Kg
Molybdenum	0.15 mg/Kg
Selenium	0.5 mg/Kg
Cadmium	No data
Chromium	1.0 mg/Kg
Fluoride	1.0 mg/Kg
Lithium	0.1 mg/Kg
Boron	2.1 mg/Kg
Nickel	0.5 mg/Kg
Vanadium	0.1 mg/Kg

Calculated Fatty Acid Composition as Fed	
Saturated Fats C12:0 and less	0.01%
Myristic Acid 14:0	0.06%
Palmitic Acid 16:0	1.75%
Stearic Acid 18:0	0.92%
Palmitoleic Acid 16:1	0.08%
Oleic Acid 18:1	3.06%
Gadoleic Acid 20:1	0.04%
Linoleic Acid 18:2 n6	4.48%
a Linolenic Acid 18:3 n3	1.20%
Arachadonic Acid 20:4 n6	No data
EPA 20:5 n3	No data
DHA 22:6 n3	No data
Total n3	1.21%
Total n6	4.48%
Total Mono Unsaturated Fats	3.19%
Total Polyunsaturated Fats	5.70%
Total Saturated Fats	2.78%

Calculated data uses information from typical raw material composition. It could be expected that individual batches of diet will vary from this figure. **Diet post treatment by irradiation or autoclave could change these parameters**. We are happy to provide full calculated nutritional information for all of our products, however we would like to emphasise that these diets have been specifically designed for manufacture by Specialty Feeds.
