# Supplementary material for: Male reproductive traits are differentially affected by dietary macronutrient balance but unrelated to adiposity
Source: Nat Commun. 2023 May 4;14:2566. doi: 10.1038/s41467-023-38314-x (PMC10160019; doi:10.1038/s41467-023-38314-x)
Supplement: Supplementary file 3 — Description of Additional Supplementary Files [file 41467_2023_38314_MOESM3_ESM.pdf]

Description of Additional Supplementary Files

File Name: Supplementary data 1

Description: AIC values and model summaries for all variables

File Name: Supplementary data 2

Description: Raw data for all measured variables
