# Supplementary material for: Male reproductive traits are differentially affected by dietary macronutrient balance but unrelated to adiposity
Source: Nat Commun. 2023 May 4;14:2566. doi: 10.1038/s41467-023-38314-x (PMC10160019; doi:10.1038/s41467-023-38314-x)
Supplement: Supplementary file 5 — Reporting Summary [file 41467_2023_38314_MOESM5_ESM.pdf]

nature portfolio

Corresponding author(s): Taylor Pini

Last updated by author(s): Apr 17, 2023

Reporting Summary

Nature Portfolio wishes to improve the reproducibility of the work that we publish. This form provides structure for consistency and transparency in reporting. For further information on Nature Portfolio policies, see our Editorial Policies and the Editorial Policy Checklist.

Statistics

For all statistical analyses, confirm that the following items are present in the figure legend, table legend, main text, or Methods section.

n/a	Confirmed	
[ ]	[x]	The exact sample size (n) for each experimental group/condition, given as a discrete number and unit of measurement
[ ]	[x]	A statement on whether measurements were taken from distinct samples or whether the same sample was measured repeatedly
[ ]	[x]	The statistical test(s) used AND whether they are one- or two-sided<br>*Only common tests should be described solely by name; describe more complex techniques in the Methods section.*
[x]	[ ]	A description of all covariates tested
[x]	[ ]	A description of any assumptions or corrections, such as tests of normality and adjustment for multiple comparisons
[ ]	[x]	A full description of the statistical parameters including central tendency (e.g. means) or other basic estimates (e.g. regression coefficient) AND variation (e.g. standard deviation) or associated estimates of uncertainty (e.g. confidence intervals)
[ ]	[x]	For null hypothesis testing, the test statistic (e.g. F, t, r) with confidence intervals, effect sizes, degrees of freedom and P value noted<br>*Give P values as exact values whenever suitable.*
[x]	[ ]	For Bayesian analysis, information on the choice of priors and Markov chain Monte Carlo settings
[x]	[ ]	For hierarchical and complex designs, identification of the appropriate level for tests and full reporting of outcomes
[ ]	[x]	Estimates of effect sizes (e.g. Cohen's d, Pearson's r), indicating how they were calculated

Our web collection on statistics for biologists contains articles on many of the points above.

Software and code

Policy information about availability of computer code

Data collection	FACSDiva (v 9.0), Zeiss Zen (blue edition, v3.4)
Data analysis	FlowJo software (v 10.7.2), ImageJ freeware (version 2.1.0), LightCycler 480 software (v 1.5.1.62), GraphPad Prism software (v 9.0.1), R (v 4.1.0)

For manuscripts utilizing custom algorithms or software that are central to the research but not yet described in published literature, software must be made available to editors and reviewers. We strongly encourage code deposition in a community repository (e.g. GitHub). See the Nature Portfolio guidelines for submitting code & software for further information.

Data

Policy information about availability of data

All manuscripts must include a data availability statement. This statement should provide the following information, where applicable:

- Accession codes, unique identifiers, or web links for publicly available datasets
- A description of any restrictions on data availability
- For clinical datasets or third party data, please ensure that the statement adheres to our policy

All raw data has been provided in the supplementary files.

Human research participants

Policy information about studies involving human research participants and Sex and Gender in Research.

Reporting on sex and gender	N/A
Population characteristics	N/A
Recruitment	N/A
Ethics oversight	N/A

Note that full information on the approval of the study protocol must also be provided in the manuscript.

Field-specific reporting

Please select the one below that is the best fit for your research. If you are not sure, read the appropriate sections before making your selection.

[x] Life sciences [ ] Behavioural & social sciences [ ] Ecological, evolutionary & environmental sciences

For a reference copy of the document with all sections, see nature.com/documents/nr-reporting-summary-flat.pdf

Life sciences study design

All studies must disclose on these points even when the disclosure is negative.

Sample size	Two studies in flies have used the NGF to assess paternal effects, the 1st using n=15 with 20 diets (Functional Ecology 2016. 30:1675-1686), and the 2nd using n=20 with 15 diets (Proceedings B 2017. 284:20171492). To be able to detect comparable effect sizes, similar sample sizes were required for the mouse study, but were more limited by ethical considerations compared to invertebrate work.
Data exclusions	No data exclusion.
Replication	Both biological and technical replicates were employed across analyses and there were no concerns that findings were not reproducible. N value (number of males) per group for each assessment was: body weight = 12, body composition = 12, reproductive organ weights = 12, testicular histology = 6, testicular gene expression = 6 (all samples assessed in duplicate technical replicates), 8-OHdG ELISA = 5 (all samples assessed in duplicate technical replicates), sperm motility = 6, sperm concentration = 6, sperm morphology = 6, sperm LIVE/DEAD stain = 6, sperm H2DCFDA stain = 6, LC-MS/MS steroid hormones = 6.
Randomization	Following an acclimation period, each cage of 3x males was randomly assigned to a diet treatment by one operator.
Blinding	Analysis of testicular architecture (Johnsen scoring) was performed by a blinded observer (i.e. blinded to group allocation during data collection), with samples prepared by another team member. For the two other subjective measures (sperm motility, sperm morphology), the same observer was responsible for preparing and assessing samples and so could not be blinded to group allocation. All other measures used were not subjective, thus blinding was not used.

Reporting for specific materials, systems and methods

We require information from authors about some types of materials, experimental systems and methods used in many studies. Here, indicate whether each material, system or method listed is relevant to your study. If you are not sure if a list item applies to your research, read the appropriate section before selecting a response.

Materials & experimental systems

n/a	Involved in the study	
[ ]	[x]	Antibodies
[x]	[ ]	Eukaryotic cell lines
[x]	[ ]	Palaeontology and archaeology
[ ]	[x]	Animals and other organisms
[x]	[ ]	Clinical data
[x]	[ ]	Dual use research of concern

Methods

n/a	Involved in the study	
[x]	[ ]	ChIP-seq
[ ]	[x]	Flow cytometry
[x]	[ ]	MRI-based neuroimaging

Antibodies

Antibodies used	8-OHdG HRP conjugated antibody, supplied as part of the 8-OHdG ELISA kit (Abcam, ab201734) and used at a 1:100 dilution (manufacturer's instructions)
Validation	Not required to be validated by species as this antibody targets a DNA modification which is not species dependent. Advertised for use with cell lysates.

Animals and other research organisms

Policy information about studies involving animals; ARRIVE guidelines recommended for reporting animal research, and Sex and Gender in Research

Laboratory animals	Four-week-old male C57BL/6J (JAX strain code 000664) mice, housed with a 12 hour light/dark cycle at 20-24 degrees celsius and 40-70% humidity.
Wild animals	No wild animals were used in the study.
Reporting on sex	Male focused study - only male mice were employed.
Field-collected samples	No field collected samples were used in the study.
Ethics oversight	The University of Sydney Animal Ethics Committee approved all procedures (project number 2019/1610).

Note that full information on the approval of the study protocol must also be provided in the manuscript.

Flow Cytometry

Plots

Confirm that:

- [x] The axis labels state the marker and fluorochrome used (e.g. CD4-FITC).
- [x] The axis scales are clearly visible. Include numbers along axes only for bottom left plot of group (a 'group' is an analysis of identical markers).
- [x] All plots are contour plots with outliers or pseudocolor plots.
- [x] A numerical value for number of cells or percentage (with statistics) is provided.

Methodology

Sample preparation	Immediately following sacrifice, sperm were isolated. Both cauda epididymides were isolated and transferred to a Petri dish containing warmed 600µL Ham's F10 medium (with 25mM HEPES and 1mM L-glutamine, catalogue number 12390-035, Thermo Fisher Scientific, Riverstone, Australia) supplemented with 1mg/mL polyvinyl alcohol (87-90% hydrolyzed, average molecular weight 30,000 – 70,000), referred to subsequently as Ham's F10 + PVA. Epididymides were cut 5 times with a sterile scalpel blade and the tissue incubated for 15 min at 37°C. Tissues were discarded and concentration of the isolated spermatozoa was determined using a Neubauer haemocytometer. Samples were subsequently diluted to 20 x 106 spermatozoa/mL with Ham's F10 + PVA and maintained at 37°C.<br>Viability was assessed using the LIVE/DEAD fixable red dead cell stain (Thermo Fisher Scientific) as per the manufacturer's directions. Briefly, a 50µL aliquot of samples diluted to 20x106 spermatozoa/mL as described above was further diluted to 2x106spermatozoa/mL with phosphate buffered saline (PBS) supplemented with 1mg/mL polyvinyl alcohol, referred to subsequently as PBS + PVA. One microlitre of stain was added and samples were incubated at 37°C for 30 min, then washed with 500µL PBS + PVA (600xg, 2 min). The resultant pellet was fixed with 10% neutral buffered formalin for 15 min, washed with 1mL PBS + PVA (600xg, 2 min) and stored at 4ºC in the dark until assessment (up to 4 days later).<br>Reactive oxygen species production was assessed using 2',7'-dichlorofluorescein diacetate (H2DCFDA). A 100µL aliquot of samples diluted to 20x106 spermatozoa/mL as described above was stained (final concentration 5µM) at 37°C for 30 min. Excess stain was removed by centrifuging samples (600xg, 2 min) and resuspending in fresh Ham's F10 + PVA. Samples were assessed for baseline ROS production immediately following stain loading and after a further 1 h incubation period. At each time point, an aliquot was counterstained with propidium iodide (PI; final concentration 6µM) for 5 min prior to assessment to discriminate the viable population.
Instrument	Becton Dickinson LSRFortessa X-20
Software	FACSDiva (v 9.0), FlowJo software (v 10.7.2)
Cell population abundance	Sperm accounted for ~50-90% of total events (based on forward vs side scatter), single cells accounted for >80% of the sperm population (based on FSC area vs FSC height). Identification of sperm was checked by back gating stained cells to original population.
Gating strategy	All samples were gated firstly on the basis of forward and side scatter to isolate spermatozoa from debris, and subsequently on the basis of forward scatter area and height to isolate single cells. For the fixable viability stain, a 610/10 BP histogram was

used to determine the proportion of viable (unstained) spermatozoa. For H2DCFDA, the population of single cells was further gated based on a 610/10 BP histogram to discriminate the viable (PI negative) population and subsequently measure median 525/50 BP detector fluorescence.

☒ Tick this box to confirm that a figure exemplifying the gating strategy is provided in the Supplementary Information.
